# Supplementary material for: Allosteric Binding Sites of Aβ Peptides on the Acetylcholine Synthesizing Enzyme ChAT as Deduced by In Silico Molecular Modeling
Source: Int J Mol Sci. 2022 May 28;23(11):6073. doi: 10.3390/ijms23116073 (PMC9181666; doi:10.3390/ijms23116073)
Supplement: Supplementary file 1 [file ijms-23-06073-s001.zip › ijms-1696212-supplementary.pdf]

International Journal of *Molecular Sciences*

Article

Allosteric Binding Sites of Aβ Peptides on the Acetylcholine Synthesizing Enzyme ChAT as Deduced by In Silico Molecular Modeling

Anurag TK Baidya [1,†], Amit Kumar [2,†], Rajnish Kumar [1,2,*,‡] and Taher Darreh-Shori [2,*,‡]

[1] Department of Pharmaceutical Engineering & Technology, Indian Institute of Technology (B.H.U.), Varanasi-221005 (U.P.), India; anuragtkbaidya.rs.phe20@itbhu.ac.in
[2] Division of Clinical Geriatric, Center for Alzheimer Research, Department of Neurobiology, Care Sciences and Society, Karolinska Institutet, NEO, 141 52 Stockholm, Sweden; amit.kumar@ki.se
* Correspondence: rajnish.phe@iitbhu.ac.in (R.K.); taher.darreh-shori@ki.se (T.D.-S.)
† These authors contributed equally to this work.
‡ These authors share the seniority on this manuscript.

Supplementary Materials

Citation: Baidya, A.T.; Kumar, A.; Kumar, R.; Darreh-Shori, T. Allosteric Binding Sites of Aβ Peptides on the Acetylcholine Synthesizing Enzyme ChAT as Deduced by In Silico Molecular Modeling. *Int. J. Mol. Sci.* **2022**, *23*, 6073. https://doi.org/10.3390/ijms23116073

Academic Editor: Maria Laura Giuffrida

Received: 8 April 2022
Accepted: 24 May 2022
Published: 28 May 2022

Publisher's Note: MDPI stays neutral with regard to jurisdictional claims in published maps and institutional affiliations.

Copyright: © 2022 by the authors. Licensee MDPI, Basel, Switzerland. This article is an open access article distributed under the terms and conditions of the Creative Commons Attribution (CC BY) license (https://creativecommons.org/licenses/by/4.0/).

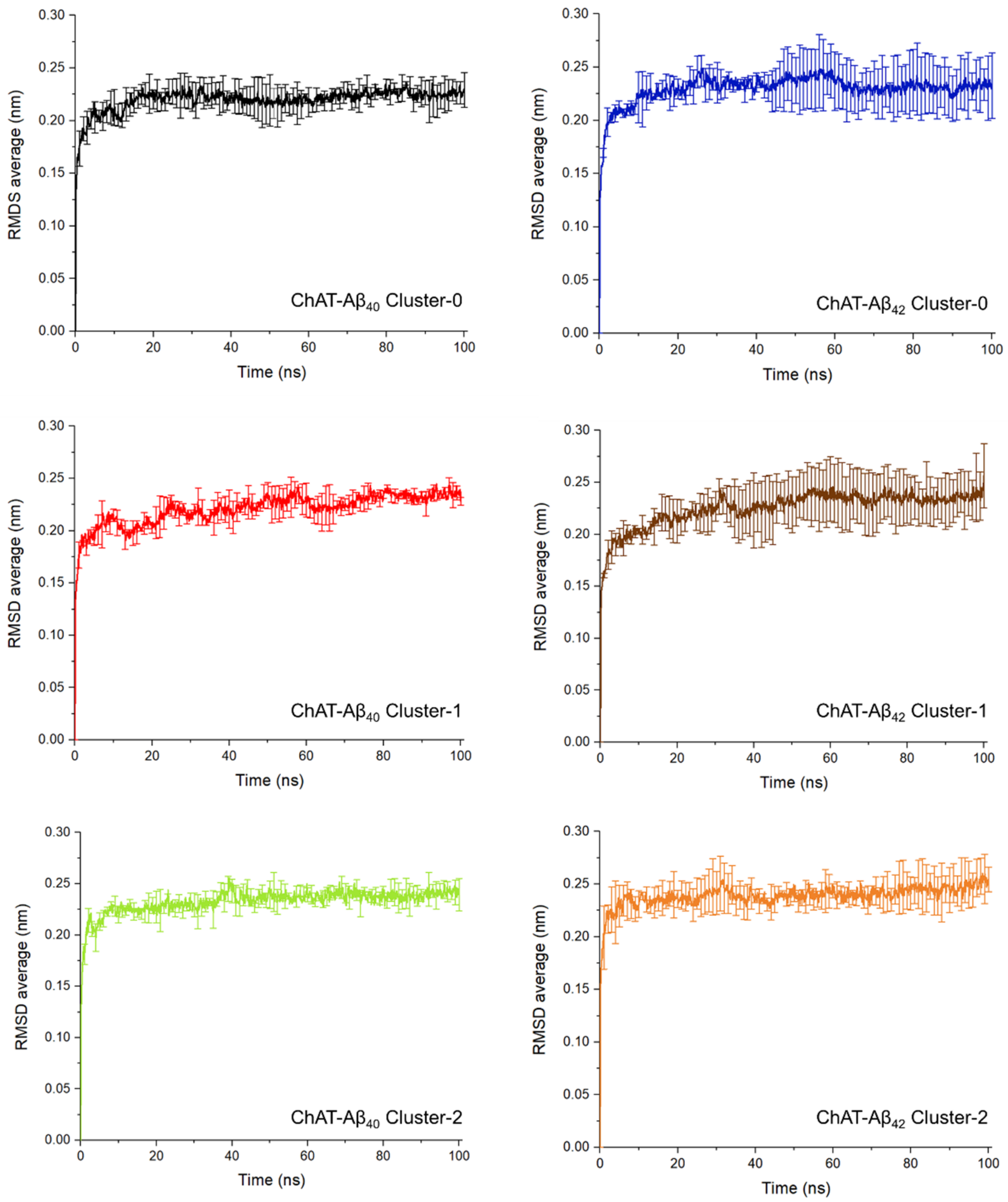

Figure S1. Average root mean square deviation (RMSD) for ChAT protein fit to backbone. Error bars represent the standard deviation (**± SD)** around the average RMSD values for three independent 100 ns MD simulations.

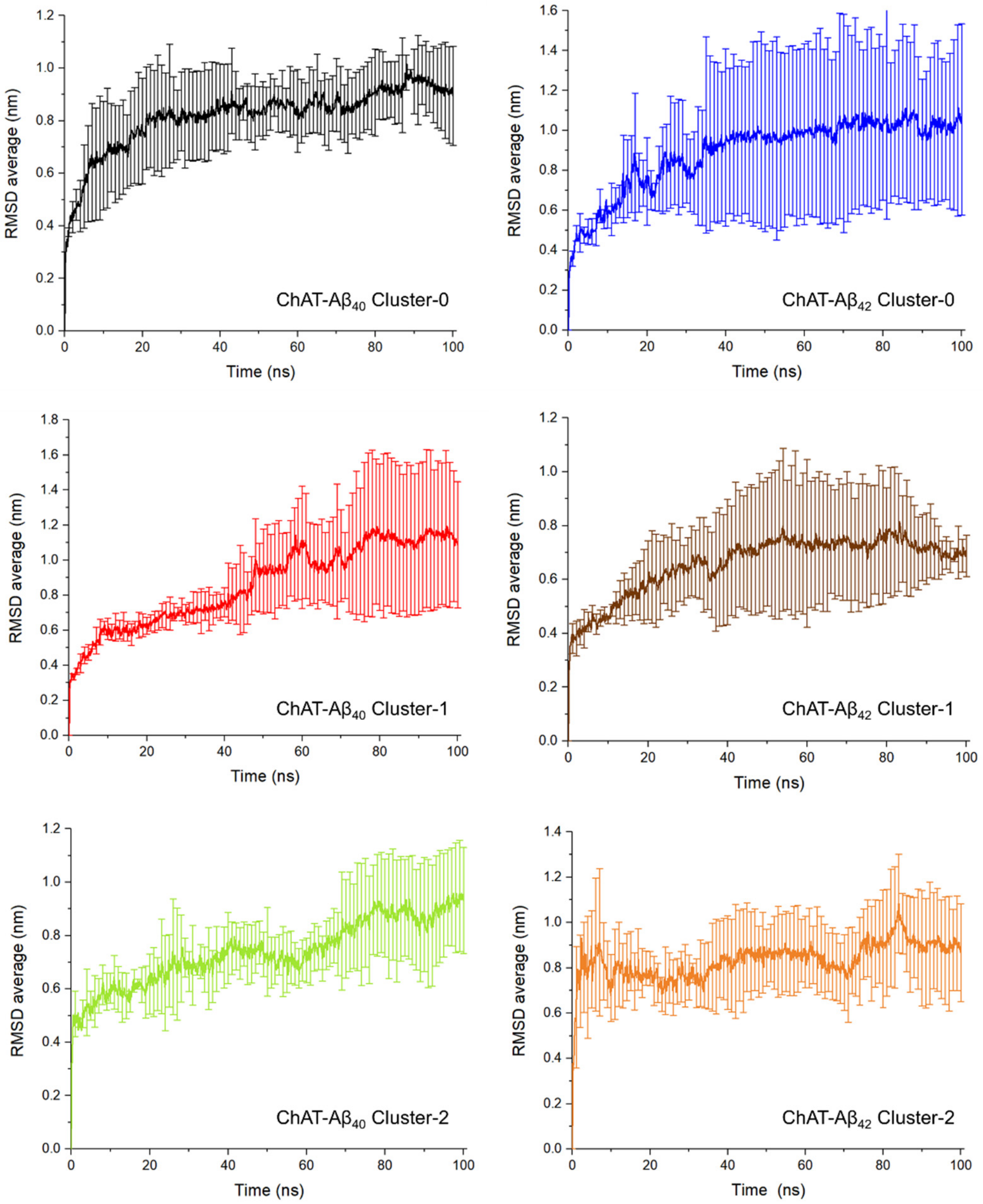

Figure S2. Average root mean square deviation (RMSD) for Aβ peptides fit to backbone. Error bars represent the standard deviation (**± SD)** around the average RMSD values for three independent 100 ns MD simulations.

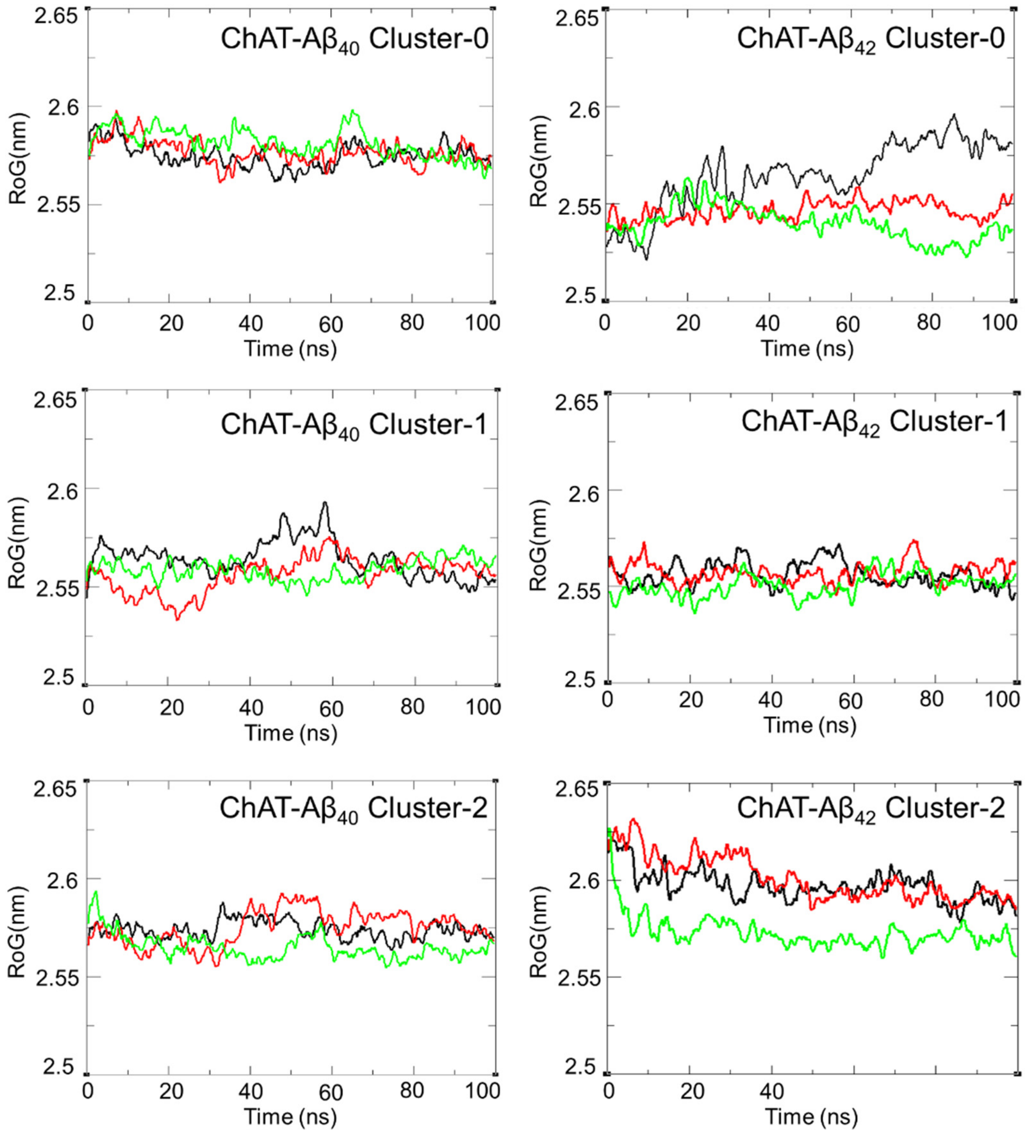

Figure S3. Radius of gyration (RoG) for the total complex system (ChAT-Aβ) during 100 ns simulations for all the triplicate runs. Each color represents the result from one of the three independent simulations. Simulation-1: Black; Simulation-2: Red; Simulation-3: Green.

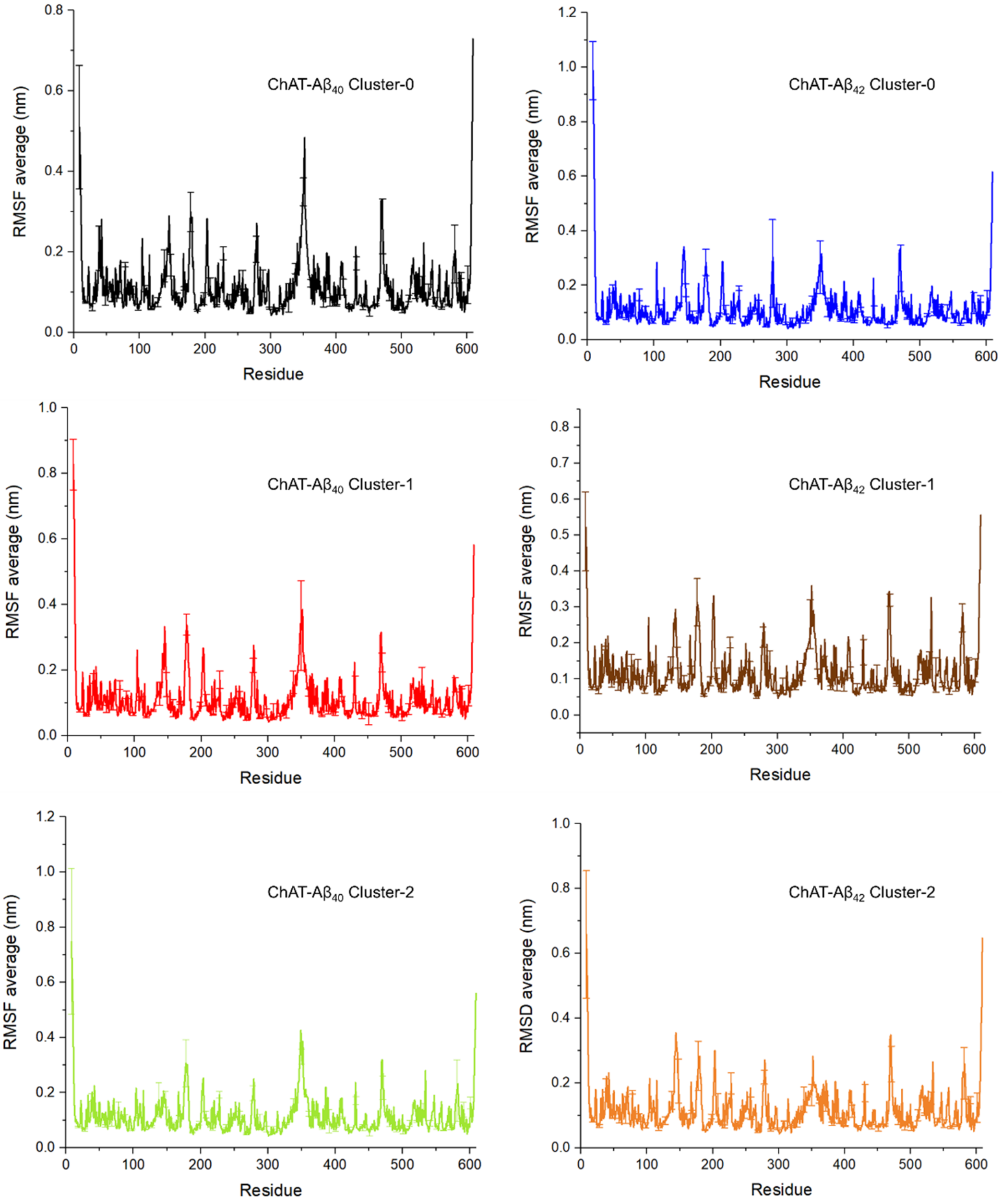

Figure S4. Average root mean square fluctuation (RMSF) of the whole ChAT protein. Error bars represent the standard deviation (**± SD)** around the average RMSF values for three independent 100 ns MD simulations.

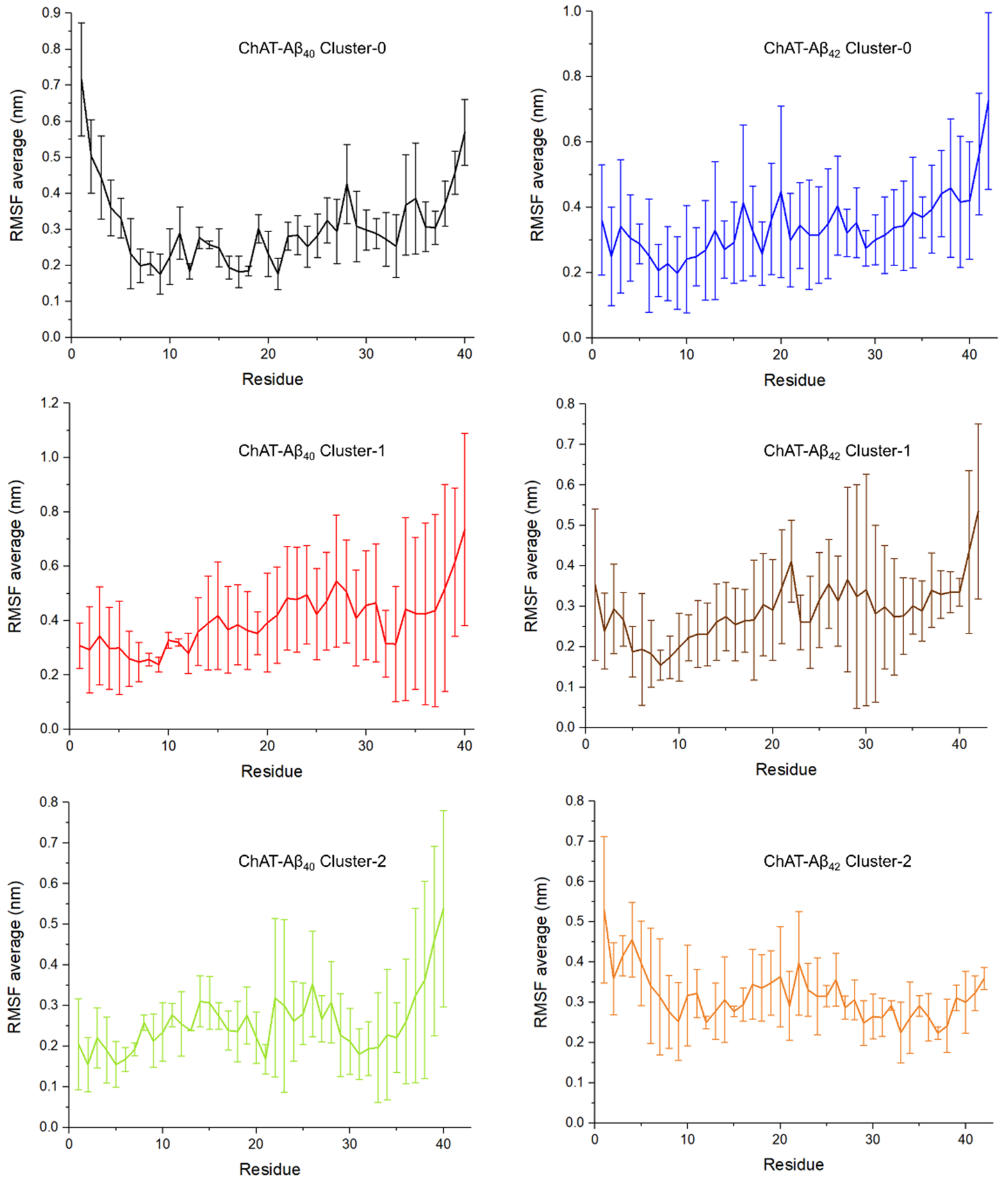

Figure S5. Average root mean square fluctuation (RMSF) of the whole Aβ peptides. Error bars represent the standard deviation (**± SD)** around the average RMSF values for three independent 100 ns MD simulations.

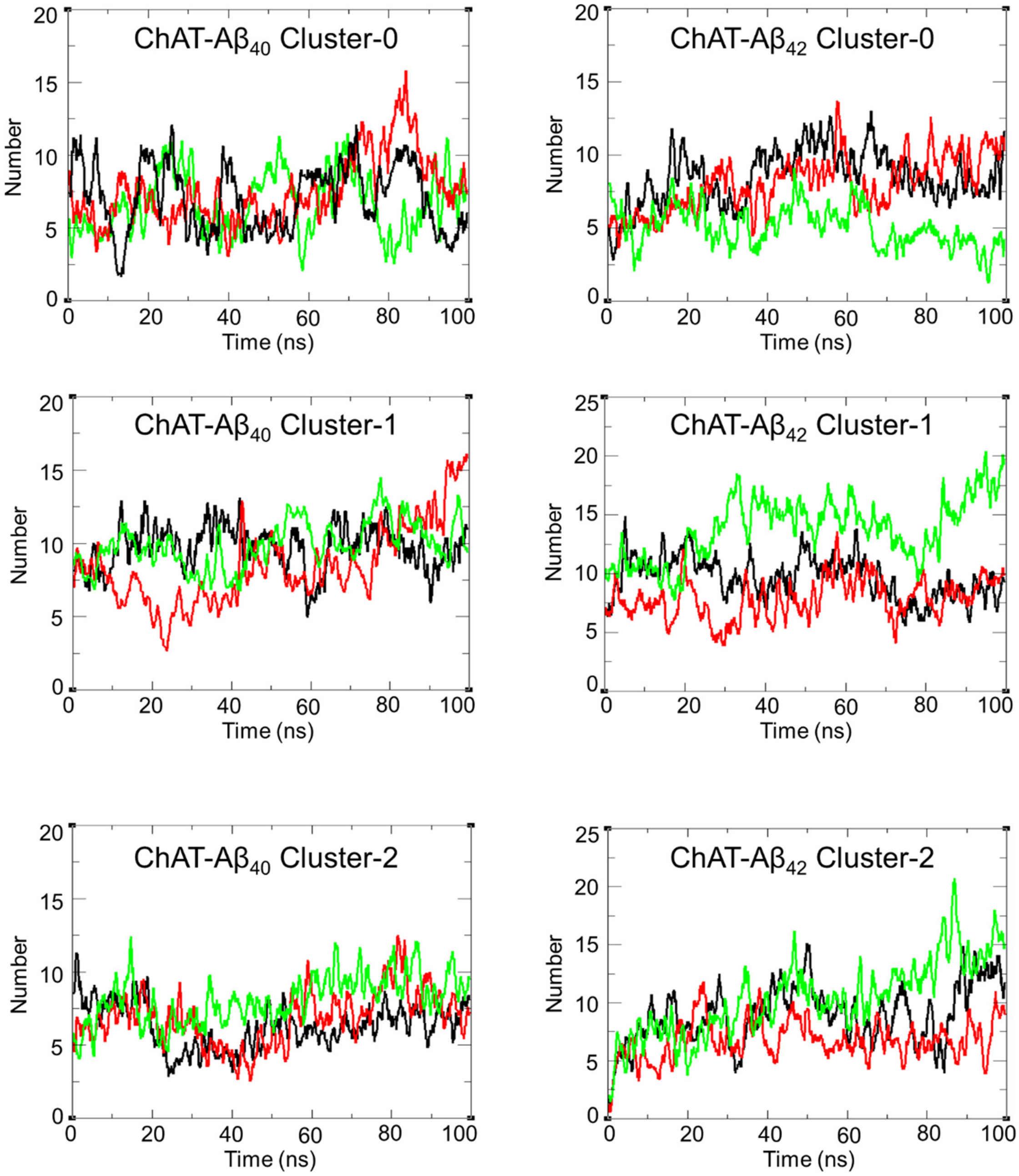

Figure S6. Hydrogen bonding landscape analysis of pairs within 0.35 nm for ChAT-Aβ complex during 100 ns simulation trajectory for all the triplicate runs. Each color represents the result from one of the three independent simulations. Simulation-1: Black; Simulation-2: Red; Simulation-3: Green.

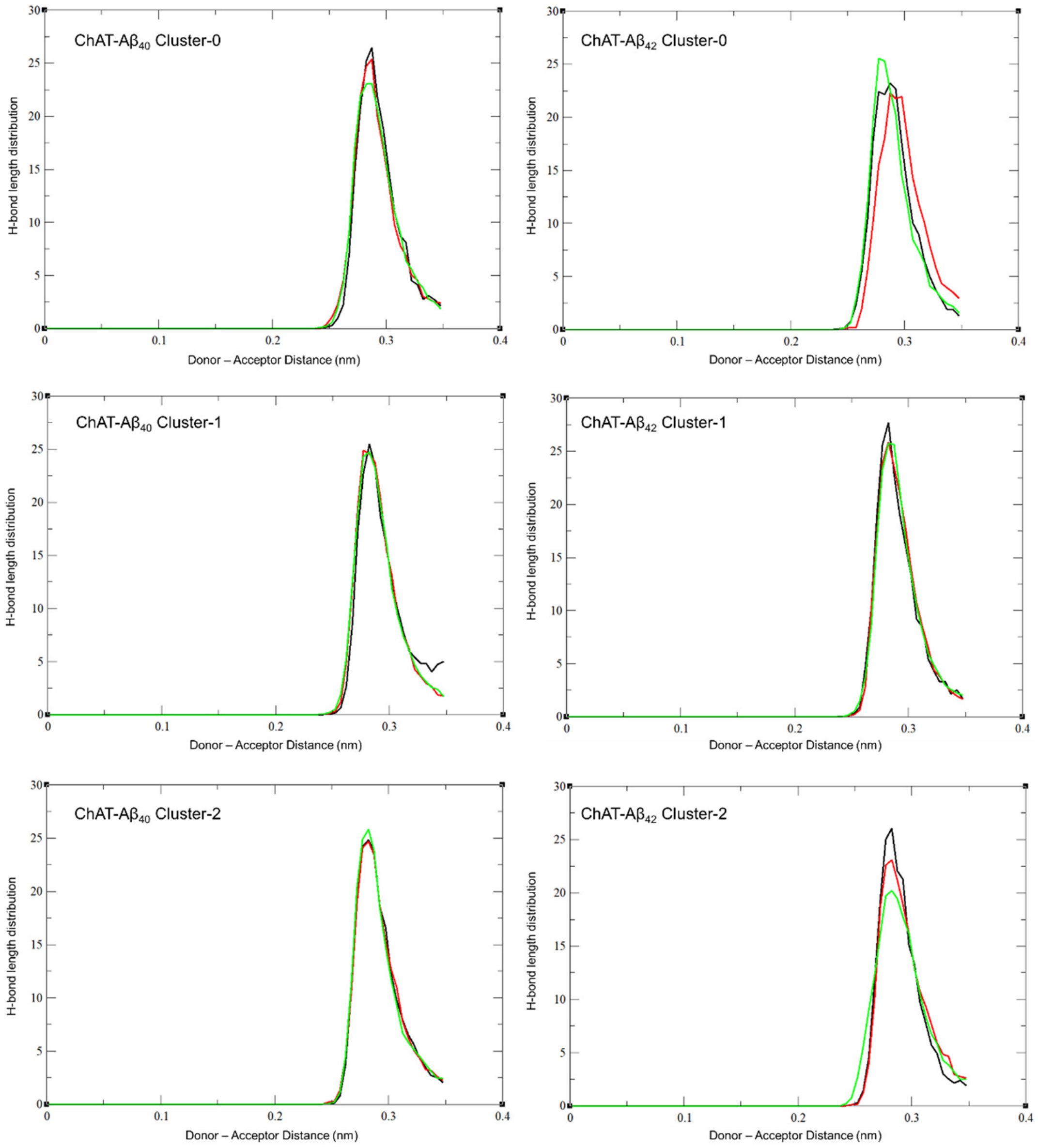

Figure S7. Average hydrogen-bond distance around the cut-off for donor–acceptor distance that was set at 0.35 nm for ChAT-Aβ complex during 100 ns simulation trajectory for all the triplicate runs. Each color represents the result from one of the three independent simulations. Simulation-1: Black; Simulation-2: Red; Simulation-3: Green.

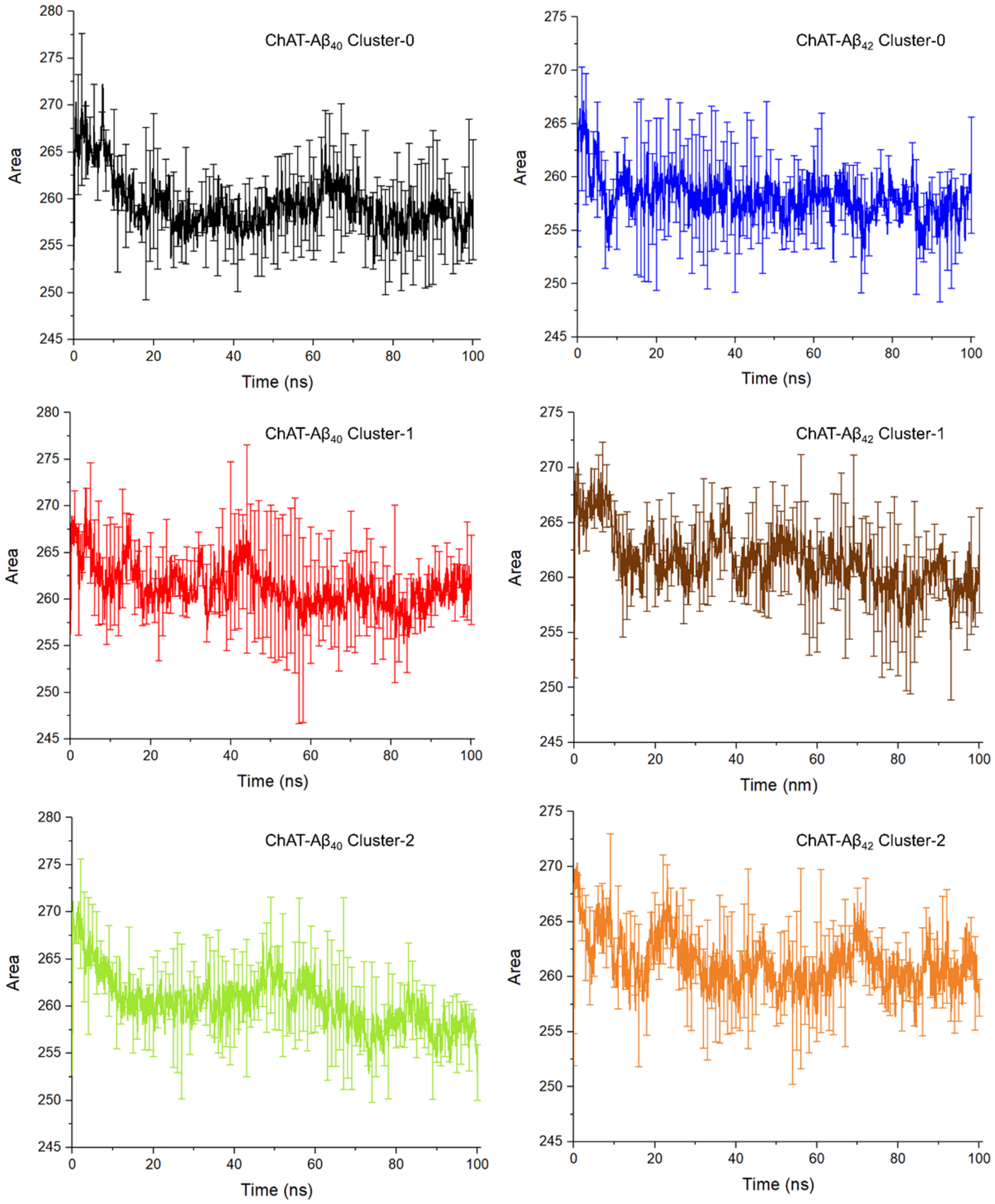

Figure S8. Average of the change in total solvent accessible surface area (SASA) of the ChAT protein, corresponding to the simulation time-period of the complex system. Error bars represent the standard deviation (± SD) around the average SASA values for three independent 100 ns MD simulations.

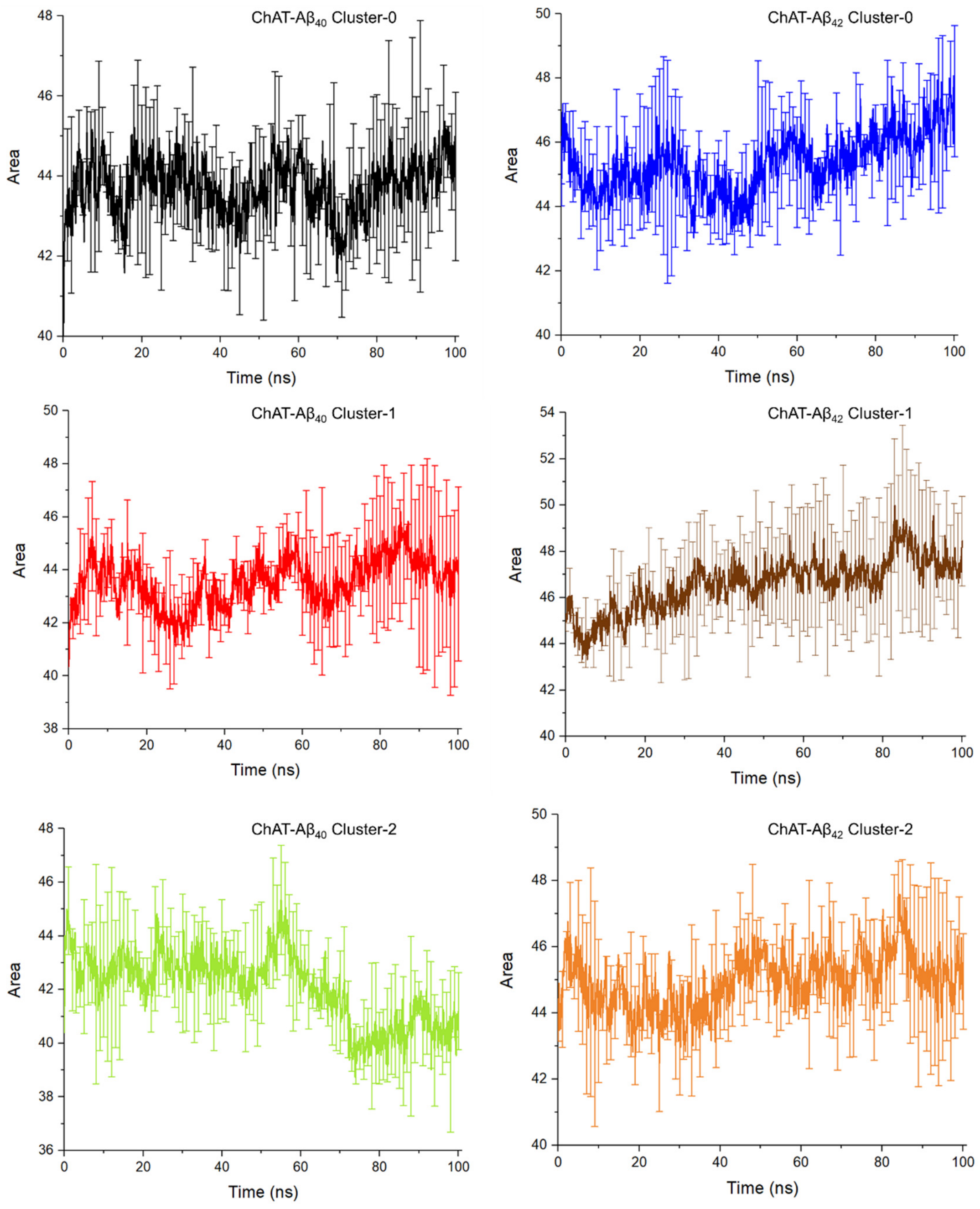

Figure S9. Average of the change in total solvent accessible surface area (SASA) of the Aβ peptides, corresponding to the simulation time-period of the complex system. Error bars represent the standard deviation (± SD) around the average SASA values for three independent 100 ns MD simulations.

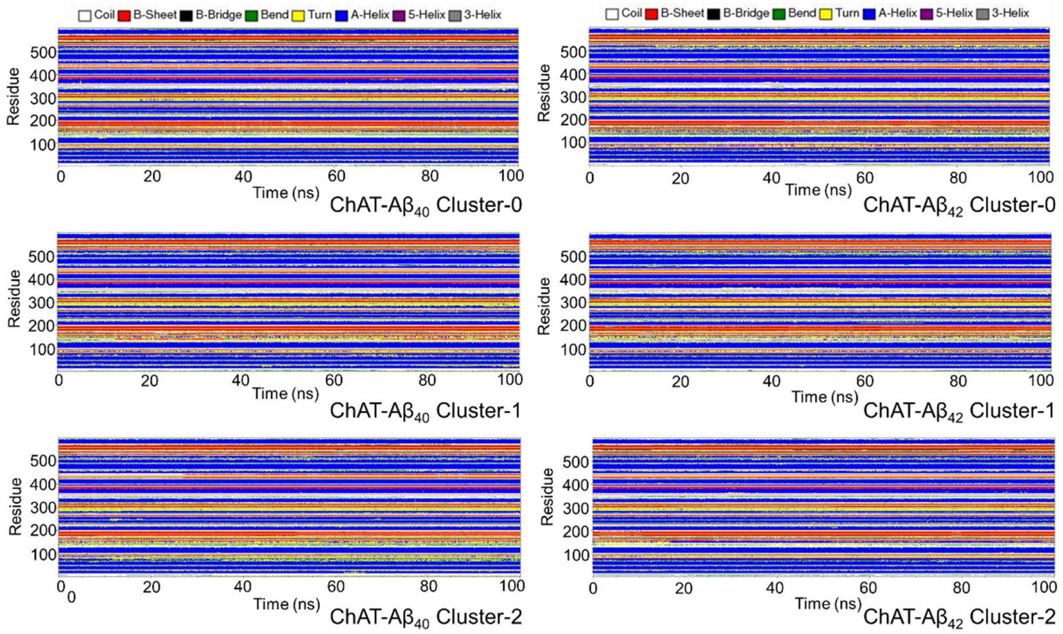

Figure S10. Time evolution of the secondary structural elements of ChAT protein from the clusters. The analyses show that the ChAT protein maintains the alpha helices throughout the simulation time, indicating that ChAT retains its quaternary folding structure and remains stable during the simulation without much of conformational changes due to the interaction with Aβ peptides.

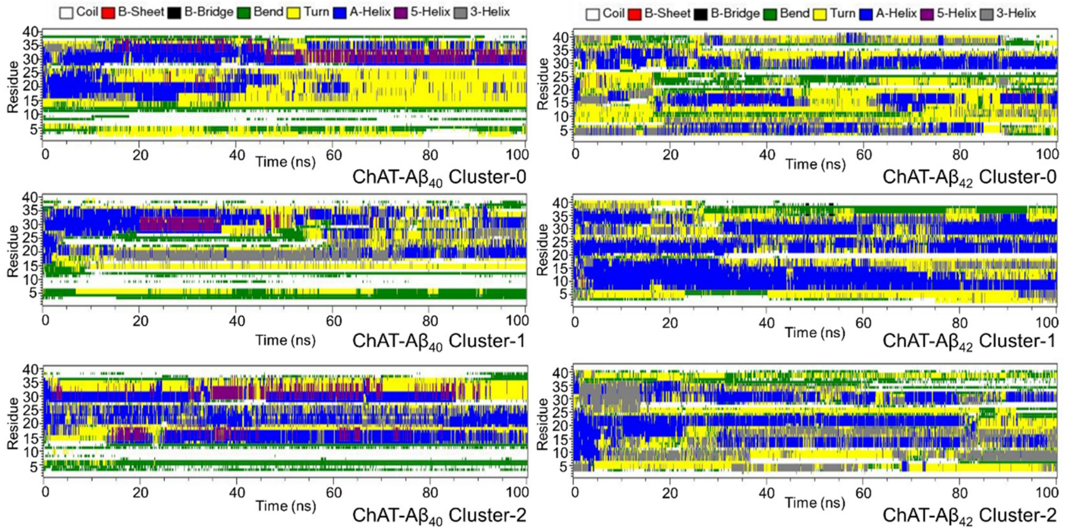

Figure S11. Time evolution of the secondary structural elements of Aβ peptides from the clusters. The analyses show that the structure of the Aβ peptides is constantly changing with time throughout the simulation, which may reflect the more occurrence of turns and coils upon its interaction with ChAT protein.

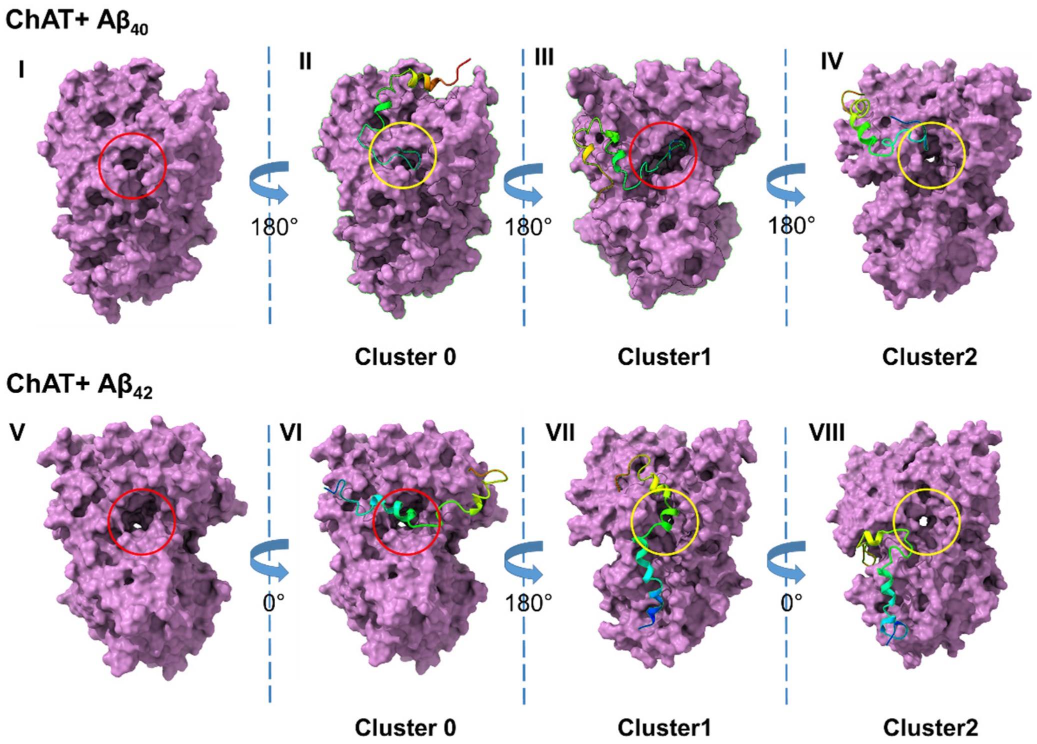

Figure S12. The final conformation derived at the end of the MD simulation of ChAT-Aβ complexes. (I) and (V) show the 3D representation of ChAT protein, where the choline-entrance of the catalytic tunnel is indicated by *red circle*. The *yellow circle* highlights the Acetyl-CoA-entrance of the catalytic tunnel. (II), (III) and (IV) represents the ChAT-$A\beta_{40}$ complexes of cluster-0, cluster-1 and cluster-2 respectively, likewise (VI), (VII) and (VIII) represents the ChAT-$A\beta_{42}$ complexes of cluster-0, cluster-1 and cluster-2 respectively. From the final structure it can be observed that the Aβ peptides remains in a similarly bound fashion to the ChAT protein like it was in the most energetically stable minima structure.
